# Gestational diabetes mellitus (GDM) in the first-reported pregnancy modifies association between interpregnancy-weight-change and GDM risk in the subsequent pregnancy

**DOI:** 10.1016/j.xagr.2026.100648

**Published:** 2026-04-15

**Authors:** Til B. Basnet, Christina R. Dantam, Charles T. Dupont, Eric S. Torstenson, Guanchao Wang, Elizabeth A. Jasper, Alison Eastman, Alexandra C. Sundermann, Etoi A. Garrison, Sarah S. Osmundson, Digna R. Velez Edwards, Zhiguo Zhao, Ayush Giri

**Affiliations:** 1 Division of Quantitative and Clinical Sciences, Department of Obstetrics and Gynecology, Vanderbilt Health, Nashville, TN (Basnet, Jasper, Sundermann, Osmundson, Edwards, and Giri); 2 Kirk Kerkorian School of Medicine, University of Nevada, Las Vegas, NV (Dantam); 3 Department of Biostatistics, Vanderbilt Health, Nashville, TN (Dupont, Wang, and Zhao); 4 Department of Biomedical Informatics, Vanderbilt Health, Nashville, TN (Torstenson and Edwards); 5 Department of Obstetrics and Gynecology, Vanderbilt Health, Nashville, TN (Eastman); 6 Division of Maternal and Fetal Medicine, Department of Obstetrics and Gynecology, Vanderbilt Health, Nashville, TN (Garrison); 7 Division of Epidemiology, Department of Medicine, Vanderbilt Health, Nashville, TN (Giri)

**Keywords:** gestational diabetes mellitus, interpregnancy-weight-change, electronic health record, effect measure modification, absolute risk

## Abstract

**Background:**

Weight change between pregnancies affects gestational diabetes mellitus (GDM) risk in subsequent pregnancies. However, it is unclear whether this impact differs based on the individual’s GDM status in a prior pregnancy.

**Objectives:**

To evaluate whether prior GDM status modifies the association between interpregnancy-weight-change and GDM risk in a subsequent pregnancy.

**Study Design:**

Using Vanderbilt Health's de-identified electronic health record, we identified 4574 individuals with at least 2 recorded pregnancies, with information available on GDM status and body mass index (BMI) at GDM screening. We computed interpregnancy-weight-change as the change in BMI at GDM screening between 2 consecutive-reported pregnancies. Using multivariable linear probability and binomial regression models, we evaluated the association between interpregnancy-weight-change and GDM risk in the second pregnancy, overall, and stratified by GDM status in the first pregnancy. We tested for effect measure modification by calculating the interaction contrast (IC) across the risk differences (RDs). We reported predicted risks for GDM, RD, and Differences in RD in the subsequent pregnancy between patients with and without GDM in their first-recorded pregnancy.

**Results:**

On average, patients gained weight (BMI increased by 0.9 kg/m^2^) between pregnancies and were more likely to have GDM in their second-recorded pregnancy (6.0% vs 4.4%). Individuals with GDM in their first pregnancy were more likely to have GDM in the second pregnancy compared to individuals without GDM in the first pregnancy (30.2% vs 5.0%). The excess risk of GDM for individuals who gained 5 BMI kg/m^2^ (∼29 pounds [lbs] in patients with BMI of 27.1 kg/m^2^ and height of 5 feet 4 inches) was substantially higher in those with GDM in their first pregnancy (RD=28.0%; 95% CI=19.1%, 36.9%) compared to those without GDM in their first pregnancy (RD=5.6%; 95% CI=3.8%, 7.4%). The IC was 22.4% (95% CI=13.4%, 31.5%; *P*<.001) across the RDs,. Similarly, the impact of weight loss (2 BMI units ∼ 12 lbs) on GDM risk reduction in the subsequent pregnancy was greater in individuals with prior GDM in their first pregnancy (RD=−3.9%; 95% CI=−0.7%, −7.2%), than among individuals without prior GDM in their first pregnancy (RD=−1.0%; 95% CI=−0.1%, −1.9%), with some evidence for effect measure modification (IC=−2.9%; 95% CI=−6.3%, 0.5%; *P*=.091). However, the predicted risk of subsequent GDM in women with prior history of GDM remains high (17.9% risk with 12 lbs weight loss and 15.4% with 29 lbs weight loss) despite substantial risk reduction.

**Conclusion:**

Interpregnancy BMI increase is associated with an elevated risk of GDM in subsequent pregnancies for all individuals but is substantially pronounced in individuals with a history of GDM. Individuals with prior GDM may mitigate their risk through weight loss, but not sufficiently to reduce it to levels similar to those without GDM in their previous pregnancy. In counseling patients with a history of GDM considering a subsequent pregnancy, it is important to encourage weight loss for various reasons, while also emphasizing that the risk of GDM remains high even with weight loss.

**AJOG Global Reports at a Glance**

**Why was this study conducted?**

To evaluate whether the effect of interpregnancy-weight-change on GDM risk in the subsequent pregnancy is modified by prior GDM status.

**Key Findings?**

Gaining weight between pregnancies increases the risk of GDM in the second pregnancy, more for individuals who had GDM in their first pregnancy than those who didn't. Conversely, losing weight reduces this risk more for individuals with prior GDM than those without; however, the absolute risk of GDM remains high despite weight loss and risk reduction.

**What does this add to what is known?**

Obesity in the first pregnancy modifies the association between interpregnancy-weight-change and GDM status in the subsequent pregnancy.

Added: Individuals with GDM in their previous pregnancy may benefit substantially from interpregnancy weight loss and may be more adversely affected by weight gain in terms of GDM risk in their subsequent pregnancy, compared to individuals without GDM in the prior pregnancy. However, even with substantial weight loss and risk reduction for GDM recurrence in patients with prior GDM, the absolute risk of GDM in the subsequent pregnancy remains high.

## Introduction

Gestational diabetes mellitus (GDM) is characterized by hyperglycemia first recognized or diagnosed after 24 weeks of gestation.^1^ In the United States, its incidence has risen from 6.0% in 2016 to 8.3% in 2021.^2^ GDM is a significant predictor of adverse maternal and fetal outcomes, including pre-eclampsia, preterm delivery, shoulder dystocia, macrosomia, and type 2 diabetes mellitus (T2DM) later in life.^1^^,^^3^, ^4^, ^5^, ^6^

Preconception health is a crucial period for addressing modifiable risk factors, such as maternal BMI, to reduce the likelihood of GDM.^7^, ^8^, ^9^ Maternal obesity and prior history of GDM are established risk factors for subsequent GDM.^10^^,^^11^ Interpregnancy weight gain is associated with an increased risk of GDM in individuals with,^12^^,^^13^ or without a previous history of GDM,^14^ or in a combined population.^15^^,^^16^ However, it remains unclear whether the effects of interpregnancy weight gain or weight loss on GDM risk in a future pregnancy are modified by GDM status from a prior pregnancy.

Leveraging electronic health record (EHR) data to identify individuals with multiple pregnancies at Vanderbilt Health (VH), our study addresses an important gap in the literature: Does the association between interpregnancy-weight-change and risk of GDM in the subsequent pregnancy differ between individuals with a history of GDM and those without?

## Methods

### Study population

**Figure 1:**
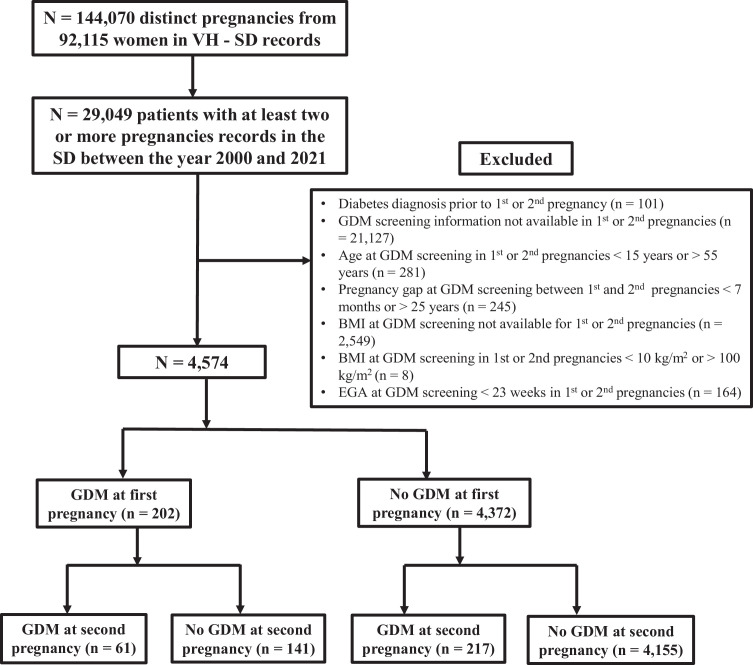
Participants’ flowchart to determine the sample size for the current analysis of gestational diabetes mellitus in the second recorded pregnancy. BMI, body mass index; EGA, estimated gestational age; GDM, gestational diabetes mellitus; VH-SD, Vanderbilt Health Synthetic Derivative. Figure shows inclusion and exclusion criteria and the number of individuals that were excluded to reach the final analytic number for primary analysis which included women with 2 or more pregnancies recorded in the VH-SD with information available on GDM screening and BMI information on the first 2 recorded consecutive pregnancies.

Using the Synthetic Derivative (SD),^17^^,^^18^ a deidentified mirror of the EHR system at VH, we considered patients with singleton pregnancies who sought care at the VH between the years 2000 and 2021. We included women with 2 or more recorded pregnancies and indexed the first 2 recorded pregnancies as the basis for evaluating GDM. We then excluded individuals based on key exclusion criteria such as if they had a diagnosis of diabetes before the first or second pregnancy and those who did not have information on GDM screening and BMI on either of the pregnancies (detailed in Figure 1). The study received a determination of non-human subjects upon review by the VH Center Institutional Review Board.

### GDM ascertainment

Providers at VH predominantly use the 2-step screening method for GDM diagnosis.^19^ Briefly, pregnant individuals receive a 1-hour screening 50-gram GCT typically between 24 and 28 weeks of gestation. Per local practice, individuals with glucose <140 mg/dL screen negative for GDM, while those with glucose ≥140 mg/dL undergo a subsequent fasting 3-hour 100-gram OGTT. A diagnosis of GDM is conferred if 2 or more abnormal OGTT results are reached using the following Carpenter Coustan thresholds: fasting ≥95 mg/dL, 1 hour ≥180 mg/dL, 2 hour ≥155, and 3 hour ≥140 mg/dL. Individuals with a fasting glucose of >126 mg/dL or a GCT glucose level of >200 mg/dL at the time of screening were also considered to have GDM. Individuals who received screening for GDM on or after 23 weeks of gestation were included in the primary analysis to allow for retention of individuals who may have been scheduled for the 24-week period but were likely seen earlier to accommodate patient provider schedules.

### Patient characteristics and estimation of interpregnancy-weight-change

To ensure consistency of measurements at uniform time points between 2 consecutive recorded pregnancies per individual, we defined interpregnancy-weight-change as the difference between BMI values recorded during the GDM screening visits of the first 2 recorded pregnancies. We further extracted information on self-reported race/ethnicity status, mother’s age, and gestational age at GDM screening, and computed the number of years between the GDM screening visits across the 2 index pregnancies (pregnancy gap).

### Statistical analysis

We compared characteristics of patients by GDM status in the second pregnancy, stratified by their GDM status in the first pregnancy. Continuous variables were compared as medians with interquartile ranges (IQR) using Wilcoxon Rank-Sum test. Categorical variables were compared using frequencies and percentages with chi-squared test, between GDM groups during the first pregnancy, and between GDM groups during the second pregnancy within each stratum defined by GDM status during the first pregnancy.

We employed linear probability models to assess the association between interpregnancy-weight-change and GDM risk in the second pregnancy, while also examining the influence of prior GDM status. These models included interpregnancy-weight-change (continuous), GDM status at first pregnancy, and their interaction terms. Covariates in the model included BMI at GDM screening in the first pregnancy (continuous), race and ethnicity, age at GDM screening in the second pregnancy (linear), pregnancy gap (linear), and estimated gestational ages at respective GDM screening for both pregnancies (linear).

When predicting the risk of GDM in the second pregnancy (hereafter referred to as second-GDM) based on interpregnancy-weight-change, all other factors were held constant at their median for continuous variables, and at the mode/largest denomination for nominal categorical variables. The predicted risks were illustrated graphically, and risk differences (RD) were calculated to compare weight loss (BMI decreases of 2 or 5 units) and weight gain (BMI increases of 2 or 5 units) against no weight change, including 95% confidence intervals and *P*-values. For reference, for a female of average height (5 feet 4 inches) with a starting BMI of 27.1 kg/m^2^ (median cohort BMI), a 2-unit and 5-unit change in BMI corresponds to a weight change of 12 lbs and 29 lbs, respectively. We assessed evidence of effect measure modification by contrasting risk differences for weight change between individuals with GDM in their first pregnancy (hereafter referred to as first-GDM) and those without and presented the difference in risk differences as the interaction contrast (IC). To align findings with existing literature, interpregnancy-weight-changes were categorized into Weight Loss (BMI change <−1 kg/m^2^), Stable Weight (−1≤ BMI-change ≤1 kg/m^2^), and Weight Gain (BMI change >1 kg/m^2^). All analyses were repeated accordingly.

In sensitivity analyses, to assess the robustness of the findings from the linear probability models, , we present risk predictions juxtaposing estimates from linear probability, logistic, and probit regression techniques to model the risk of second-GDM. We also estimated predicted risks and average RDs from binomial probability models, removing individuals who received GDM screening before 24 weeks in either of the 2 pregnancies and in individuals whose pregnancy gap was less than 3 or 5 years. Finally, to evaluate whether BMI at GDM screening in the first pregnancy impacts findings related to interpregnancy-weight-change and subsequent GDM risk, we also estimated predicted probability of GDM in second pregnancy as a function of interpregnancy BMI change, while fixing BMI in the first GDM screening at 20, 30 and 35 kg/m^2^, stratified by first-GDM. A 2-sided *P*-value of <.05 was considered statistically significant. All statistical analyses and estimation of summary data for participant characteristics were conducted using R software, version 4.3.

## Results

Out of 144,070 distinct pregnancies from 92,115 individuals, 29,049 had 2 or more pregnancies during the study period. After excluding individuals based on eligibility criteria and missingness (Figure 1), a total of 4574 individuals with information available on BMI, GDM status on the first 2 recorded pregnancies, and estimated gestational age at GDM screening ≥23 weeks in both pregnancies were included in the analysis.

Four percent of individuals had gestational diabetes in their first-recorded pregnancy, and 6.0% had GDM in their second-recorded pregnancy. Approximately 65.5% identified as non-Hispanic White, followed by 14.5% non-Hispanic Black, 10.7% Hispanic, 6.1% non-Hispanic Asian, and 3.2% identified as other race categories. Individuals who identify as Asian were most likely to have first-GDM (9.3%), followed by Hispanic (5.1%), with similar trends in the second pregnancy (*P*<.001). Individuals who had first-GDM were more likely to have second-GDM (30.2%) compared to individuals who did not have first-GDM (5.0%, *P*<.001). Compared to individuals without first-GDM, those with GDM tended to be older (median age: 27.2 vs 28.6 years, *P*<.001), and have higher BMI in their first (27.1 vs 30.3 kg/m^2^*, P*<.001) and second pregnancy (28.1 vs 31.1 kg/m^2^*, P*<.001). Patients without first-GDM were likely to gain more weight with a median increase of BMI of 0.9 kg/m^2^ compared to 0.6 kg/m^2^ for those with GDM (*P*=.004) (Table 1 and Supplementary Tables 1–2).

**Table 1:** Characteristics of patients stratified by gestational diabetes mellitus status in first recorded pregnancy.

	No GDM during first pregnancy				GDM during first pregnancy				
	GDM status during second pregnancy				GDM status during secon^d^ pregnancy				
	No GDM^a^	GDM^a^	Combined^a^	*P*^b^	No GDM^a^	GDM^a^	Combined^a^	*P*^c^	*P*^d^
N (%)	4155 (95.0%)	217 (5.0%)	4372 (95.6%)		141 (69.8%)	61 (30.2%)	202 (4.4%)		<.001
Race/Ethnicity				<.001				.518	<.001
Non-Hispanic White	2749 (95.9%)	118 (4.1%)	2867 (95.7%)		94 (72.3%)	36 (27.7%)	130 (4.3%)		
Non-Hispanic Black	623 (96.1%)	25 (3.9%)	648 (97.7%)		10 (66.7%)	5 (33.3%)	15 (2.3%)		
Non-Hispanic Asian	227 (89.0%)	28 (11.0%)	255 (90.7%)		18 (69.2%)	8 (30.8%)	26 (9.3%)		
Hispanic	425 (91.8%)	38 (8.2%)	463 (94.9%)		14 (56.0%)	11 (44.0%)	25 (5.1%)		
Others	131 (94.2%)	8 (5.8%)	139 (95.9%)		5 (83.3%)	1 (16.7%)	6 (4.1%)		
Age at GDM screening									
During first pregnancy	27.1 (23.1, 30.4)	28.0 (23.9, 31.3)	27.2 (23.1, 30.5)	.011	28.6 (25.1, 32.8)	28.4 (25.4, 32.2)	28.6 (25.2, 32.4)	.970	<.001
During secon^d^ pregnancy	30.0 (26.0, 33.1)	31.5 (27.2, 34.7)	30.0 (26.1, 33.2)	<.001	31.2 (27.6, 35.5)	32.0 (28.4, 35.6)	31.3 (27.9, 35.5)	.616	<.001
Estimated gestational age									
During first pregnancy	28.4 (25.9, 29.4)	28.0 (25.7, 29.4)	28.3 (25.9, 29.4)	.925	27.7 (25.2, 29.4)	27.9 (25.2, 29.4)	27.7 (25.2, 29.4)	.802	.244
During secon^d^ pregnancy	27.1 (25.0, 29.4)	26.9 (24.7, 29.3)	27.1 (25.0, 29.4)	.550	27.5 (24.6, 29.4)	25.4 (24.0, 29.1)	27.0 (24.3, 29.4)	.096	.808
BMI at GDM screening									
During first pregnancy	26.9 (24.3, 30.9)	28.8 (26.0, 33.0)	27.1 (24.4, 31.0)	<.001	29.4 (25.5, 34.7)	31.4 (28.3, 34.5)	30.3 (26.1, 34.7)	.219	<.001
During secon^d^ pregnancy	27.9 (24.9, 32.2)	31.8 (28.4, 36.0)	28.1 (25.0, 32.5)	<.001	29.9 (25.6, 35.2)	33.3 (29.0, 36.3)	31.1 (26.4, 35.6)	.017	<.001
Interpregnancy age gap	2.4 (1.8, 3.3)	3.0 (1.9, 4.2)	2.4 (1.8, 3.4)	<.001	2.3 (1.7, 3.0)	2.7 (1.8, 3.9)	2.4 (1.7, 3.3)	.034	.911
BMI change (continuous)^e^	0.8 (−0.3, 2.1)	2.0 (0.5, 3.8)	0.9 (−0.2, 2.2)	<.001	0.3 (−1.2, 1.8)	1.2 (0.0, 2.6)	0.6 (−0.9, 2.2)	.003	.004
BMI change (categories)^f^				<.001				.059	<.001
Weight loss	550 (97.3%)	15 (2.7%)	565 (92.0%)		39 (79.6%)	10 (17.9%)	49 (8.7%)		
Stable weight	1694 (96.9%)	54 (3.1%)	1748 (96.1%)		52 (73.2%)	19 (26.8%)	71 (3.9%)		
Weight gain	1911 (92.8%)	148 (7.2%)	2059 (96.2%)		50 (61.0%)	32 (39.0%)	82 (3.8%)		

BMI, body mass index; GDM, gestational diabetes mellitus.

a Median (first quartile, third quartile) for continuous variables; Frequency and percentages for categorical variables. The percentages presented for combined are percentages out of the overall population.

b Comparing GDM vs no GDM during second pregnancy among those with no GDM in the first pregnancy.

c Comparing GDM vs no GDM during second pregnancy among those with GDM in first pregnancy.

d Comparing GDM vs no GDM during first pregnancy (the 2 combined columns).

e BMI changes was defined as: the difference between BMI at screening during second recorded pregnancy and BMI at screening during first recorded pregnancy.

f BMI changes categories were defined as: stable weight (1≤ BMI change ≤1); weight loss (BMI change <−1); weight gain (BMI change >1).

**Figure 2:**
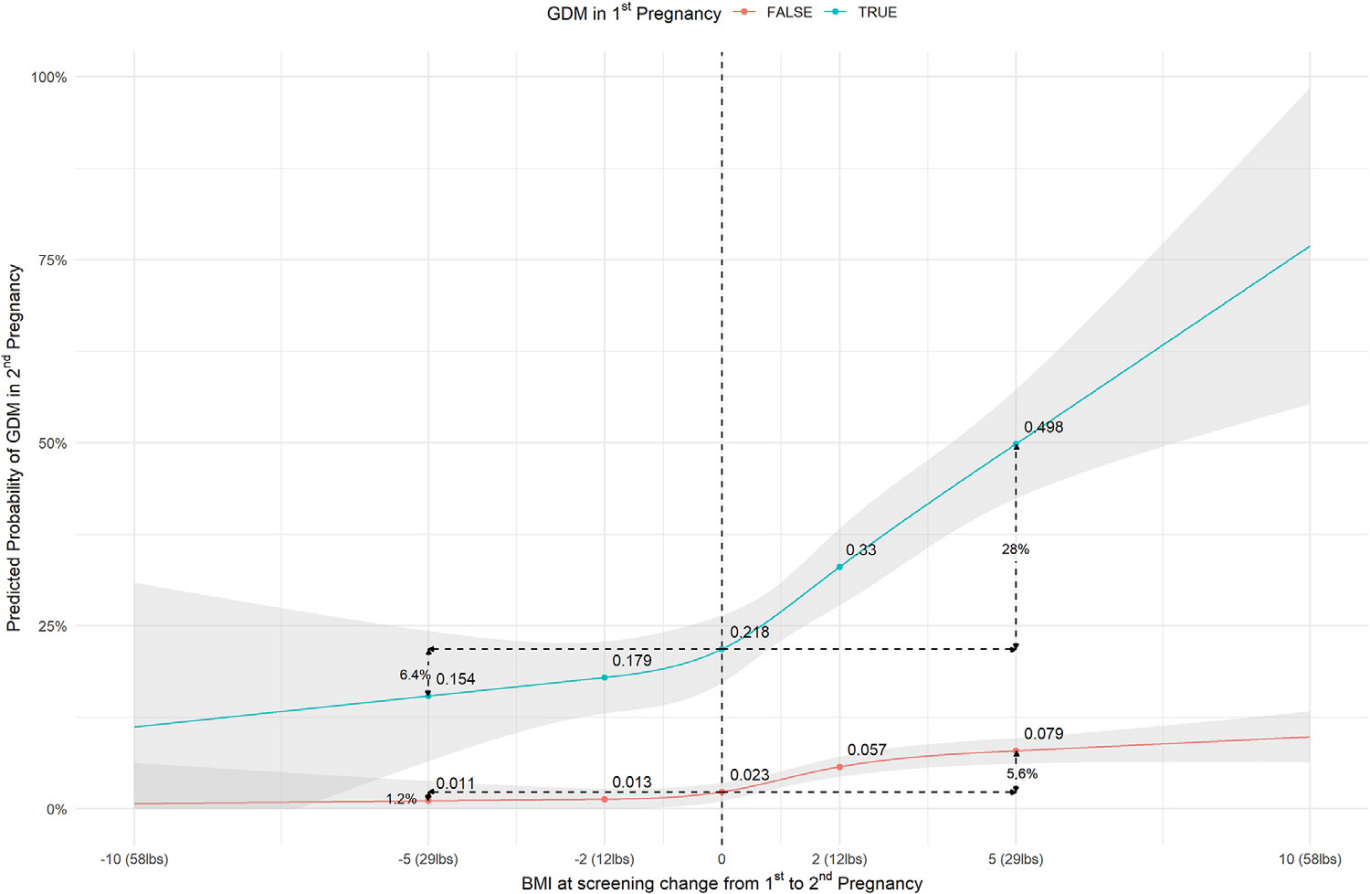
Predicted probability of gestational diabetes mellitus (GDM) in the second-recorded pregnancy associated with change in body mass index from first to second recorded pregnancies stratified by GDM status at screening in the first-recorded pregnancy. BMI, body mass index; GDM, gestational diabetes status; Figure shows predicted absolute risk/ predicted probability of GDM in the second recorded pregnancy (y-axis) stratified by GDM status in the first recorded pregnancy, modeled using linear probability models for the following specifications: mother’s age at screening of second recorded pregnancy (30.1 years), time gap between screenings across two pregnancies (2.4 years), BMI at screening during first recorded pregnancy (27.1 kg/m^2^), race/ethnicity (Non-Hispanic White), estimated gestational ages at screening during the first (28.4 weeks) and second (27.3 weeks) recorded pregnancies, and the interaction term between first-pregency-GDM status and interpregnancy-weight-change. The value of 0 at the x-axis denotes no change in BMI from the first- to second-recorded pregnancy at the GDM screening visit, whereas a positive number denotes weight gain (in BMI units), and a negative number denotes weight loss (in BMI units). Risk differences were computed, taking contrasts of the predicted risks at various x-axis values.

**Figure 3:**
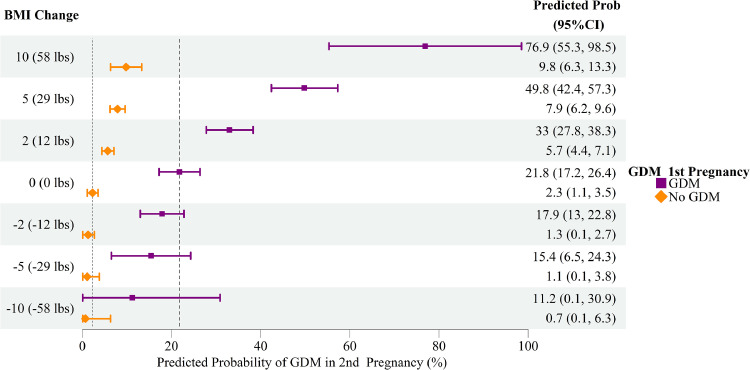
Forest plot showing predicted probability of gestational diabetes mellitus (GDM) in the second-recorded pregnancy associated with change in body mass index from first to second recorded pregnancies stratified by GDM status at screening in the first-recorded pregnancy. BMI: body mass index; GDM: gestational diabetes status; Figure shows predicted absolute risk/ predicted probability of GDM in the second recorded pregnancy (y-axis) stratified by GDM status in the first recorded pregnancy, modeled using linear probability models for the following specifications: mother’s age at screening of second recorded pregnancy (30.1 years), time gap between screenings across two pregnancies (2.4 years), BMI at screening during first recorded pregnancy (27.1 kg/m^2^), race/ethnicity (Non-Hispanic White), estimated gestational ages at screening during the first (28.4 weeks) and second (27.3 weeks) recorded pregnancies, and the interaction term between first-pregency-GDM status and interpregnancy-weight-change. The value of 0 at the x-axis denotes no change in BMI from the first- to second-recorded pregnancy at the GDM screening visit, whereas a positive number denotes weight gain (in BMI units), and a negative number denotes weight loss (in BMI units). Risk differences were computed, taking contrasts of the predicted risks at various x-axis values.

Compared to individuals with stable weight in both pregnancies (BMI change=0), the absolute risk (AR) of GDM increased by 6.5% (predicted risk difference (RD)=6.5%; 95% CI=4.6%, 8.3%) in those who gained 5 BMI units (29 lbs), and decreased by 1.8% (RD=−1.8%; 95% CI=−4.3%, 0.8%) in those who lost 5 BMI units (Table 2). When stratified by GDM status in the first-reported pregnancy, the excess risk of GDM for individuals who gained 5 BMI units was substantially higher in those with first-GDM (RD=28.0%; 95% CI=19.1%, 36.9%) compared to those without first-GDM (RD=5.6%; 95% CI=3.8%, 7.4%) (Table 2; Figure 2). We tested for effect measure modification by calculating the difference in predicted RDs (IC=22.4%; 95% CI=13.4%, 31.5%; *P*<.001). This indicated that 22 additional cases per 100 individuals would be expected due to weight gain among individuals with prior GDM, compared with the combined effect of prior GDM alone (without weight gain) and weight gain alone among individuals without prior GDM. Similarly, the impact of weight loss (2 BMI units equivalent to 12 lbs) on GDM risk reduction in the subsequent pregnancy was greater in individuals with first-GDM (RD=−3.9%; −7.2%, −0.7%), than among individuals without first-GDM (RD=−1.0%; 95% CI=−1.9%, −0.1%), with some evidence for effect measure modification (IC=−2.9%; 95% CI=−6.3%, 0.5%; *P*=.0.091). Although interpregnancy weight loss in women with GDM in their first-recorded pregnancy led to a larger reduction in GDM risk in their subsequent pregnancy, the absolute risk of GDM remained high, with GDM risk at 17.9% (95% CI: 13.0%, 22.8%) with a 12 lbs loss, and 15.4% (95% CI: 6.5%, 24.3%) with a 29 lbs loss from the first pregnancy (Figure 2, Figure 3). In contrast, the absolute risk of GDM in the subsequent pregnancy in women without prior history of GDM was 1.3% (95% CI: 0.0%, 2.7%) with a 12 lbs loss and 1.1% (95% CI: 0.0%, 3.8%) with a 29 lbs weight loss.

**Table 2:** Differences in predicted risks of gestational diabetes mellitus at the second recorded pregnancy associated with interpregnancy change in body weight.

	Overall population		During first pregnancy among				Differences between RD in GDM and non-GDM	
			non-GDM		GDM			
Interpregnancy weight change	RD (95% CI)^a^	*P*	RD (95% CI)^a^	*P*	RD (95% CI)^a^	*P*	IC (95% CI)^a^	*P*
Continuous^b^								
−5 vs 0	−1.8% (−4.3%, 0.8%)	.187	−1.2% (−3.9%, 1.4%)	.366	−6.4% (−16.4%, 3.5%)	.204	−5.2% (−15.4%, 5.0%)	.318
−2 vs 0	−1.3% (−2.1%, −0.4%)	.004	−1% (−1.9%, −0.1%)	.027	−3.9% (−7.2%, −0.7%)	.019	−2.9% (−6.3%, 0.5%)	.091
2 vs 0	3.9% (2.6%, 5.2%)	<.001	3.4% (2.1%, 4.8%)	<.001	11.2% (4.8%, 17.6%)	.001	7.8% (1.3%, 14.3%)	.019
5 vs 0	6.5% (4.7%, 8.3%)	<.001	5.6% (3.8%, 7.4%)	<.001	28.0% (19.1%, 36.9%)	<.001	22.4% (13.4%, 31.5%)	<.001
Categories^c^								
Weight loss	−1.7% (−3.8%, 0.4%)	.121	−1.2% (−3.4%, 1%)	.291	−6.1% (−14.5%, 2.3%)	.154	−4.9% (−13.5%, 3.8%)	.268
Stable weight	*Ref*		*Ref*		*Ref*		*Ref*	
Weight gain	3.6% (2.2%, 5.1%)	<.001	3.3% (1.8%, 4.8%)	<.001	12.3% (5%, 19.6%)	.001	9% (1.6%, 16.5%)	.017

BMI, body mass index; CI, confidence interval; GDM, gestational diabetes mellitus; IC, interaction contrast; *P, P*-value; RD, risk difference.

a The RDs were calculated using predicted risks estimated from linear probability models including mother’s age at screening of second recorded pregnancy, time gap between screenings across 2 pregnancies, BMI at screening during first recorded pregnancy, race/ethnicity, estimated gestational ages at screening during the first and second recorded pregnancies, and the interaction term between first-pregency-GDM status and interpregnancy weight changes.

b Interpregnancy weight change was defined as: BMI at screening during second recorded pregnancy minus BMI at screening during first recorded pregnancy; 0 represents change in BMI=0; negative number represents weight loss; and positive number represents weight gain.

c BMI changes categories were defined as: stable weight (1≤ BMI change ≤1 kg/m^2^); weight loss (BMI change <−1 kg/m^2^); weight gain (BMI change >1 kg/m^2^).

In sensitivity analyses, categorizing interpregnancy-weight-change data as weight loss, stable weight, and weight gain led to similar findings (Table 2). Similarly, predicted probabilities and resulting predicted risk differences estimated from multivariable logistic and probit regression models were similar (Supplementary Figure 1). Furthermore, restricting analyses to individuals with a pregnancy gap of fewer than 5 years or 3 years (Supplementary Figure 2) and limiting analyses to individuals whose estimated gestational age at GDM screening was >24 weeks (Supplementary Figure 3) showed similar results, albeit with reduced precision.

Compared to those included in the main analyses, individuals who were excluded (N=24,475) were more likely to be non-Hispanic black (18.8% vs 14.5% ), less likely to be Asian (3.8% vs 6.1% ) and younger on average in their second pregnancy (28.6 vs 30.1 years), but did not differ substantially otherwise (Supplementary Table 3). Comparing individuals who had GDM screening data but were excluded due to missing BMI at 1 or more screening visits (N=2549) yielded similar results (Supplementary Table 4). Predicted risks of second-GDM by race/ethnicity status (Supplementary Figure 4) and by starting BMI in the first pregnancy (Supplementary Figure 5) showed similar patterns.

## Comment

### Principal findings

In an EHR-derived cohort of individuals with 2 or more pregnancies, we report that the effect of interpregnancy-weight-change on GDM risk in the second pregnancy is modified by whether the individual had GDM in their previous pregnancy. Patients with prior GDM had a substantially greater increase in risk of subsequent GDM when they experienced weight gain, compared to patients without prior GDM and experienced comparable weight gain. Likewise, weight loss led to a greater reduction in GDM risk in individuals who had GDM in their previous pregnancy compared to individuals who did not have GDM in their previous pregnancy and lost weight. Yet, despite a greater reduction in risk, the absolute risk of GDM in the second-recorded pregnancy in women with prior GDM remains high at 17.9% even with a 12 lbs weight loss (2 BMI units), and 15.4% with a 29 lbs weight loss (5 BMI units).

### Results in the context of what is known

In a large retrospective cohort study based on the Kaiser Permanente Southern California population, Getahun et al.^20^ reported that the risk of GDM during the second pregnancy was 41.3% among patients with a prior GDM diagnosis, compared to only 4.2% for those without. In agreement with this study and others,^21^, ^22^, ^23^ we show that individuals with first-GDM are substantially more likely to have GDM in their subsequent pregnancy (32%) compared to individuals without first-GDM (5.3%). Our estimate of GDM recurrence (32%) falls within the previously described broad range (30–80%).^6^^,^^9^^,^^23^, ^24^, ^25^, ^26^

Studies evaluating how interpregnancy-weight-change affects GDM risk in a subsequent pregnancy report either on 1) the overall impact of weight change or 2) focus on the impact of weight loss stratified by overweight/obesity status in the first pregnancy.^13^, ^14^, ^15^ Using the Norwegian birth registry data from 2006–2014, interpregnancy weight gain (defined as the difference between pre-pregnancy BMI across 2 pregnancies) in individuals without first-GDM was associated with increased risk of second-GDM in all individuals, however, weight loss was only associated with reduced risk in individuals with a BMI ≥25 kg/m^2^ in their first pregnancy.^13^ Another study comprised only individuals with a prior history of GDM from the Swedish and Norwegian medical birth registries, similarly reported a positive association between interpregnancy weight gain and GDM recurrence in all individuals; however, weight loss was associated with reduced risk, especially in individuals with BMI ≥25 kg/m^2^.^14^

Studies including individuals with and without GDM in their first pregnancies also report similar conclusions on the role of weight change on GDM risk in the second pregnancy.^12^, ^13^, ^14^, ^15^, ^16^ Relevant to the US population, a large retrospective cohort analysis in Northern California^20^ additionally reported that overweight individuals with more than 2 BMI units of weight loss between pregnancies had a relative 74% reduced odds of GDM.^15^ However, these studies do not evaluate whether first-GDM modifies the role of weight change on subsequent GDM risk.

In agreement with these studies we further expand findings in the literature to show that first-GDM modifies the association between weight change and GDM risk in the next pregnancy. We show that the excess risk of second-GDM associated with interpregnancy-weight-gain among individuals with first-GDM is substantially higher than women without first-GDM with comparable interpregnancy-weight-gain. Similarly, the benefits associated with weight loss in reducing GDM risk in the second pregnancy are also greater for individuals who had first-GDM compared to those who did not. Furthermore, we report that even with substantial weight loss and risk reduction in recurrence of GDM, the absolute risk of subsequent GDM in those with prior first-GDM remains substantially high. We demonstrate similar trends in Black, Asian and Hispanic women; however, stratification leads to a substantial reduction in precision due to smaller numbers in each of these race/ethnic groups.

### Clinical implications

Our research carries important implications for both patients and clinicians, highlighting the critical role of interpregnancy weight management in reducing the risk of recurrent GDM, and yet underscores the limits of weight loss on risk reduction. Our results indicate that even among individuals [first pregnancy BMI=27.1 kg/m^2^] who maintain a stable BMI across both pregnancies, having GDM in the previous pregnancy is associated with a greater predicted absolute risk of second-GDM (28.0%) compared to individuals who did not have first-GDM (5.6%) (Figure 2). This difference widens with interpregnancy weight gain. Substantial weight loss (a reduction of 5 BMI units in a patient with a BMI of 27.1 kg/m^2^ in their first pregnancy) in individuals with prior GDM can decrease the risk of recurrence from 21.8% to 15.4%. Although this represents a substantial decrease, the absolute risk of GDM of 15.9% is still high. The preconception period is an opportunity for primary-care clinicians to intervene, offering targeted messaging to individuals of reproductive age who are overweight/obese and particularly to those with a history of GDM. In communicating with patients, providers may need to emphasize the limits of weight loss alone in reducing the risk of GDM and additional strategies, which improve insulin sensitivity (such as physical activity), and reduce exposure to hyperglycemia (dietary intervention), also need to be prioritized to further mitigate susceptibility in this high-risk population.

### Research implications, strengths, and limitations

Research related to interpregnancy-weight-change and maternal comorbidities is primarily limited to data from national registries in European countries and large-scale Health Insurance-based studies such as Kaiser Permanente in the US. The present study highlights the usefulness and limitations of EHR data in identifying a cohort of individuals with 2 or more pregnancies, with data available on GDM screening from a single medical center, as it allows evaluation of a question that would be difficult, time-consuming, and expensive to investigate with active recruitment. The findings from this study should thus be assessed considering the following points:

1) Ideally, interpregnancy-weight-change would be computed by subtracting BMI values taken right before pregnancy. As up to 50% of pregnancies are unplanned,^27^ data on pre-pregnancy BMI is not always reliably available, especially in EHR data primarily collected to provide care. Therefore, we used the GDM screening visit across both pregnancies per individual as the index visits to anchor measurements for weight, gestational age, and other characteristics that may interfere with inferences on interpregnancy-weight-change and GDM risk. Reliant on this method, the interpregnancy-weight-change variable may not purely capture weight change prior to pregnancy. Assuming similar amounts of gestational weight change between 2 pregnancies, the interpretation of our findings may be reflective of interpregnancy-weight-change. Although extant literature on patterns of gestational weight gain across multiple pregnancies is sparse, few studies that have evaluated this show individuals who exceeded gestational weight gain according to guidelines in the first pregnancy were 5 times as likely to also exceed gestational weight gain guidelines in the second pregnancy, suggesting reduced within-person-between-pregnancy variability in gestational weight gain.^28^ While the study is not able to fully tease apart the contributions from interpregnancy-weight-change and differential gestational weight gain, it does not take away from the message that weight loss alone, and limiting gestational weight gain leading up to the time of GDM screening, can reduce GDM risk in women with prior GDM risk but that the reduction in risk is not sufficient.
2) The cohort is limited to pregnancies observed in 1 academic medical center, and we refer to these as first-recorded and second-recorded pregnancies. To reduce the possibility of missing pregnancies not observed at VH, limiting analyses to individuals who had a pregnancy gap of less than 5 years and less than 3 years showed similar findings.
3) We were unable to evaluate if the observed associations further vary by race/ethnicity status, mother’s birth status (US or foreign), or by strata of obesity in the first pregnancy. Such an investigation evaluating 3-way interactions would require sample sizes far larger than this current investigation and remains an extremely relevant topic for future research. With the lack of longitudinally linked birth registry data in the US, our study underscores the importance of utilizing EHR-based methods across institutions to evaluate these questions.

## Conclusion

The impact of interpregnancy-weight-change on GDM risk in a subsequent pregnancy is substantially greater in individuals with a history of GDM than in individuals without. While substantial weight reduction prior to GDM screening in the second pregnancy may mitigate risk of recurrence, the absolute risk of GDM recurrence remains high. In counseling patients with a history of GDM who are looking to get pregnant, the recommendation to maintain ideal body weight alone is insufficient. Placing an emphasis on the limits of what weight loss alone can achieve in reducing future risk of GDM is warranted.

## Tweetable statement

Weight loss between pregnancies greatly reduces gestational diabetes [GDM] risk in the next pregnancy in women with a history of GDM vs those w/o. Yet, recurrence risk remains high: weight loss alone is not enough!

## Ethical approval statement

This study was approved by the Vanderbilt Health Institutional Review Board.

## CRediT authorship contribution statement

**Til B. Basnet:** Writing – review & editing, Writing – original draft, Visualization, Investigation. **Christina R. Dantam:** Writing – review & editing, Writing – original draft. **Charles T. Dupont:** Writing – review & editing, Data curation. **Eric S. Torstenson:** Writing – review & editing, Data curation. **Guanchao Wang:** Writing – review & editing, Visualization. **Elizabeth A. Jasper:** Writing – review & editing. **Alison Eastman:** Writing – review & editing. **Alexandra C. Sundermann:** Writing – review & editing. **Etoi A. Garrison:** Writing – review & editing. **Sarah S. Osmundson:** Writing – review & editing. **Digna R. Velez Edwards:** Writing – review & editing. **Zhiguo Zhao:** Writing – review & editing, Writing – original draft, Visualization, Supervision, Investigation. **Ayush Giri:** Writing – review & editing, Supervision, Methodology, Investigation, Data curation, Conceptualization.

## Appendix Supplementary materials

Image, application 1

## Footnotes

Supplementary material associated with this article can be found, in the online version, at doi:10.1016/j.xagr.2026.100648.
