# Supplementary material for: Gestational diabetes mellitus (GDM) in the first-reported pregnancy modifies association between interpregnancy-weight-change and GDM risk in the subsequent pregnancy
Source: AJOG Glob Rep. 2026 Apr 15;6(2):100648. doi: 10.1016/j.xagr.2026.100648 (PMC13202284; doi:10.1016/j.xagr.2026.100648)
Supplement: Supplementary file 1 [file mmc1.docx]

**Table of Contents**

[**A.** **SUPPLEMENTARY FIGURES** 2](#_Toc222741151)

[**Figure legend and caption with description** 2](#_Toc222741152)

[**Supplementary Figure 1. Models comparing association between interpregnancy weight change and GDM risk in the second-recorded pregnancy reported as predicted probability stratified by GDM status in the first-recorded pregnancy for linear probability, logistic, and probit models.** 4](#_Toc222741153)

[**Supplementary Figure 2. Predicted probability of GDM in the second recorded pregnancy associated with interpregnancy weight change from first to second recorded pregnancies stratified by GDM status at screening in the first recorded pregnancy among individuals with interpregnancy time gap of A) less than 5 years and B) less than 3 years** 5](#_Toc222741154)

[**Supplementary Figure 3. Predicted probability of GDM in the second recorded pregnancy associated with interpregnancy weight change from first to second recorded pregnancies stratified by GDM status at screening in the first recorded pregnancy among individuals whose GDM screening visit in both pregnancies were done at >=24 weeks of pregnancy.** 6](#_Toc222741155)

[**Supplementary Figure 4. Predicted probability of gestational diabetes mellitus (GDM) across various races-ethnicities in the second-recorded pregnancy associated with change in body mass index from first to second recorded pregnancies stratified by GDM status at screening in the first-recorded pregnancy** 7](#_Toc222741156)

[**Supplementary Figure 5. Predicted probability of GDM in the second recorded pregnancy associated with interpregnancy weight change from first to second recorded pregnancies fixing BMI in the first pregnancy at 20, 30, and 35 kg/m^2^ while stratified by GDM status in the first pregnancy** 8](#_Toc222741157)

[**B.** **SUPPLEMENTARY TABLES** 9](#_Toc222741158)

[**Supplementary Table 1. Demographic characteristics of participants stratified by GDM status in the first- and second-recorded pregnancies** 9](#_Toc222741159)

[**Supplementary Table 2. Clinical and demographic characteristics of participants stratified by categorical exposure status, namely weight loss, no change, and weight gain.** 10](#_Toc222741160)

[**Supplementary Table 3. Comparison of patient characteristics: excluded vs. analyzed data sets based on BMI information** 12](#_Toc222741161)

[**Supplementary Table 4. Comparison of patient characteristics: excluded vs. analyzed data sets based on the availability of screening information** 13](#_Toc222741162)

# **SUPPLEMENTARY FIGURES**

## **Figure legend and caption with description**

**Supplementary Figure 1. Models comparing the association between interpregnancy weight change and GDM risk in the second-recorded pregnancy reported as predicted probability stratified by GDM status in the first-recorded pregnancy for linear probability, logistic, and probit models.**

GDM: Gestational diabetes status; BMI: Body mass index; LPM: Linear probability model

The predicted probabilities of GDM (y-axis) were computed as a function of various values of change in BMI from the first to second pregnancy (x-axis) from models with the following specifications: mother’s age at screening of second recorded pregnancy (30.1 years), time gap between screenings across two pregnancies (2.4 years), BMI at screening during first recorded pregnancy (27.1 kg/m^2^), race/ethnicity (Non-Hispanic White), estimated gestational ages at screening during the first (28.4 weeks) and second (27.3 weeks) recorded pregnancies, and the interaction term between 1^st^-Pregency-GDM status and interpregnancy weight change..

Change in BMI (decrease/increase) indicates the differences in BMI from the first to second-recorded pregnancies at GDM screening; Predicted probability of GDM in second pregnancy stratified by GDM in first pregnancy: Solid line = No GDM in first pregnancy; Dashed line = GDM in first pregnancy. Red line = estimates from logistic model; Green line = estimates from LPM; Blue line = estimates from probit model.

**Supplementary Figure 2. Predicted probability of GDM in the second recorded pregnancy associated with interpregnancy weight change from first to second recorded pregnancies stratified by GDM status at screening in the first recorded pregnancy among individuals with interpregnancy time gap of A) less than 5 years and B) less than 3 years**

GDM: Gestational diabetes status; BMI: Body mass index; Figure shows predicted absolute risk of GDM in the second recorded pregnancy (y-axis) stratified by GDM status in the first recorded pregnancy, modeled using linear probability models for the following specifications: mother’s age at screening of second recorded pregnancy (30.1 years), time gap between screenings across two pregnancies (2.4 years), BMI at screening during first recorded pregnancy (27.1 kg/m^2^), race/ethnicity (Non-Hispanic White), estimated gestational ages at screening during the first (28.4 weeks) and second (27.3 weeks) recorded pregnancies, and the interaction term between 1^st^-Pregency-GDM status and interpregnancy weight change. The value of 0 at the x-axis denotes no change in BMI from the first to second pregnancy, whereas a positive number denotes weight gain (in BMI units), and a negative number denotes weight loss (in BMI units). Risk differences were computed, taking contrasts of the predicted risks at various x-axis values. The graph represents predicted probabilities from models estimated restricting to women whose first and recorded pregnancies were within 5 years (left: Panel A); and within 3. Years (right: Panel B).

**Supplementary Figure 3. Predicted probability of GDM in the second recorded pregnancy associated with interpregnancy weight change from first to second recorded pregnancies stratified by GDM status at screening in the first recorded pregnancy among individuals whose GDM screening visit in both pregnancies were done at >=24 weeks of pregnancy.**

GDM: Gestational diabetes status; BMI: Body mass index; Figure shows predicted absolute risk of GDM in the second recorded pregnancy (y-axis) stratified by GDM status in the first recorded pregnancy, modeled using linear probability models for the following specifications: mother’s age at screening of second recorded pregnancy (30.1 years), time gap between screenings across two pregnancies (2.4 years), BMI at screening during first recorded pregnancy (27.1 kg/m^2^), race/ethnicity (Non-Hispanic White), estimated gestational ages at screening during the first (28.4 weeks) and second (27.3 weeks) recorded pregnancies, and the interaction term between 1^st^-Pregency-GDM status and interpregnancy weight change. The value of 0 at the x-axis denotes no change in BMI from the first to second pregnancy, whereas a positive number denotes weight gain (in BMI units), and a negative number denotes weight loss (in BMI units). Risk differences were computed, taking contrasts of the predicted risks at various x-axis values. The graph represents predicted probabilities from models estimated restricting to individuals whose GDM screening visits in both pregnancies were done at >=24 weeks of pregnancy.

**Supplementary Figure 4. Predicted probability of gestational diabetes mellitus (GDM) across various races-ethnicities in the second-recorded pregnancy associated with change in body mass index from first to second recorded pregnancies stratified by GDM status at screening in the first-recorded pregnancy**

GDM: Gestational diabetes status; BMI: Body mass index; Figure shows predicted absolute risk/ predicted probability of GDM in the second recorded pregnancy (y-axis) stratified by GDM status in the first recorded pregnancy, modeled using linear probability models for the following specifications: mother’s age at screening of second recorded pregnancy (30.1 years), time gap between screenings across two pregnancies (2.4 years), BMI at screening during first recorded pregnancy (27.1 kg/m^2^), race/ethnicity (Non-Hispanic White), estimated gestational ages at screening during the first (28.4 weeks) and second (27.3 weeks) recorded pregnancies, and the interaction term between 1^st^-Pregency-GDM status and interpregnancy weight change.. The value of 0 at the x-axis denotes no change in BMI from the first- to second-recorded pregnancy at the GDM screening visit, whereas a positive number denotes weight gain (in BMI units), and a negative number denotes weight loss (in BMI units). Risk differences were computed, taking contrasts of the predicted risks at various x-axis values.

**Supplementary Figure 5. Predicted probability of GDM in the second recorded pregnancy associated with interpregnancy weight change from first to second recorded pregnancies fixing BMI in the first pregnancy at 20, 30, and 35 kg/m^2^ while stratified by GDM status in the first pregnancy.**

GDM: Gestational diabetes status; BMI: Body mass index; Figure shows predicted absolute risk of GDM in the second recorded pregnancy (y-axis) stratified by GDM status in the first recorded pregnancy (A: GDM in the first pregnancy; B: No GDM in the first pregnancy), modeled using linear probability models while fixing BMI in the first pregnancy at 20 (red line), 30 (green line), and 35 kg/m^2^ (blue line) with the following specifications: mother’s age at screening of second recorded pregnancy (30.1 years), time gap between screenings across two pregnancies (2.4 years), race/ethnicity (Non-Hispanic White), estimated gestational ages at screening during the first (28.4 weeks) and second (27.3 weeks) recorded pregnancies, and the interaction term between 1^st^-Pregency-GDM status and interpregnancy weight change. The value of 0 at the x-axis denotes no change in BMI from the first to second pregnancy, whereas a positive number denotes weight gain (in BMI units), and a negative number denotes weight loss (in BMI units). Risk differences were computed, taking contrasts of the predicted risks at various x-axis values.

##
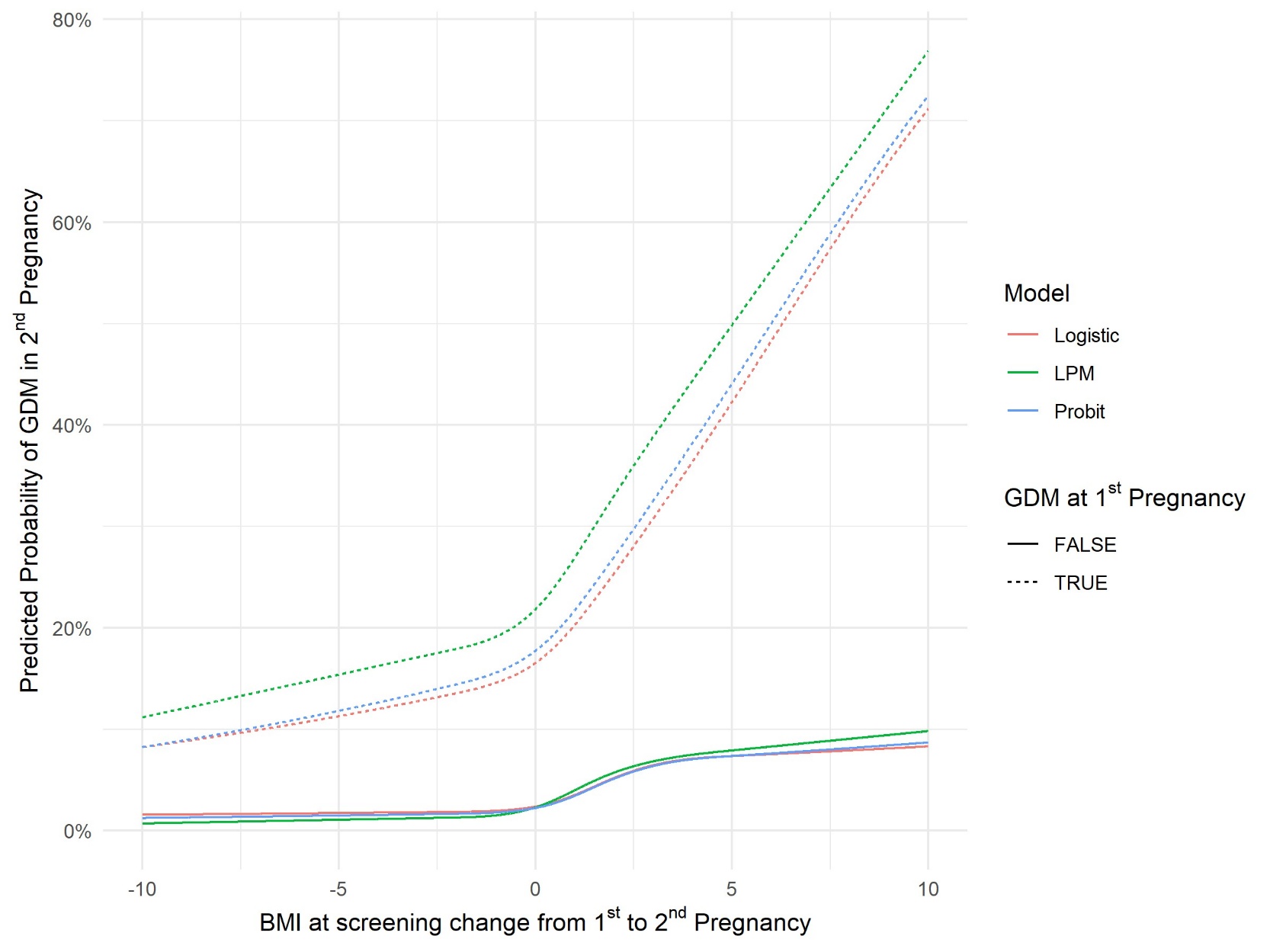
**Supplementary Figure 1. Models comparing association between interpregnancy weight change and GDM risk in the second-recorded pregnancy reported as predicted probability stratified by GDM status in the first-recorded pregnancy for linear probability, logistic, and probit models.**

## **Supplementary Figure 2. Predicted probability of GDM in the second recorded pregnancy associated with interpregnancy weight change from first to second recorded pregnancies stratified by GDM status at screening in the first recorded pregnancy among individuals with interpregnancy time gap of A) less than 5 years and B) less than 3 years**


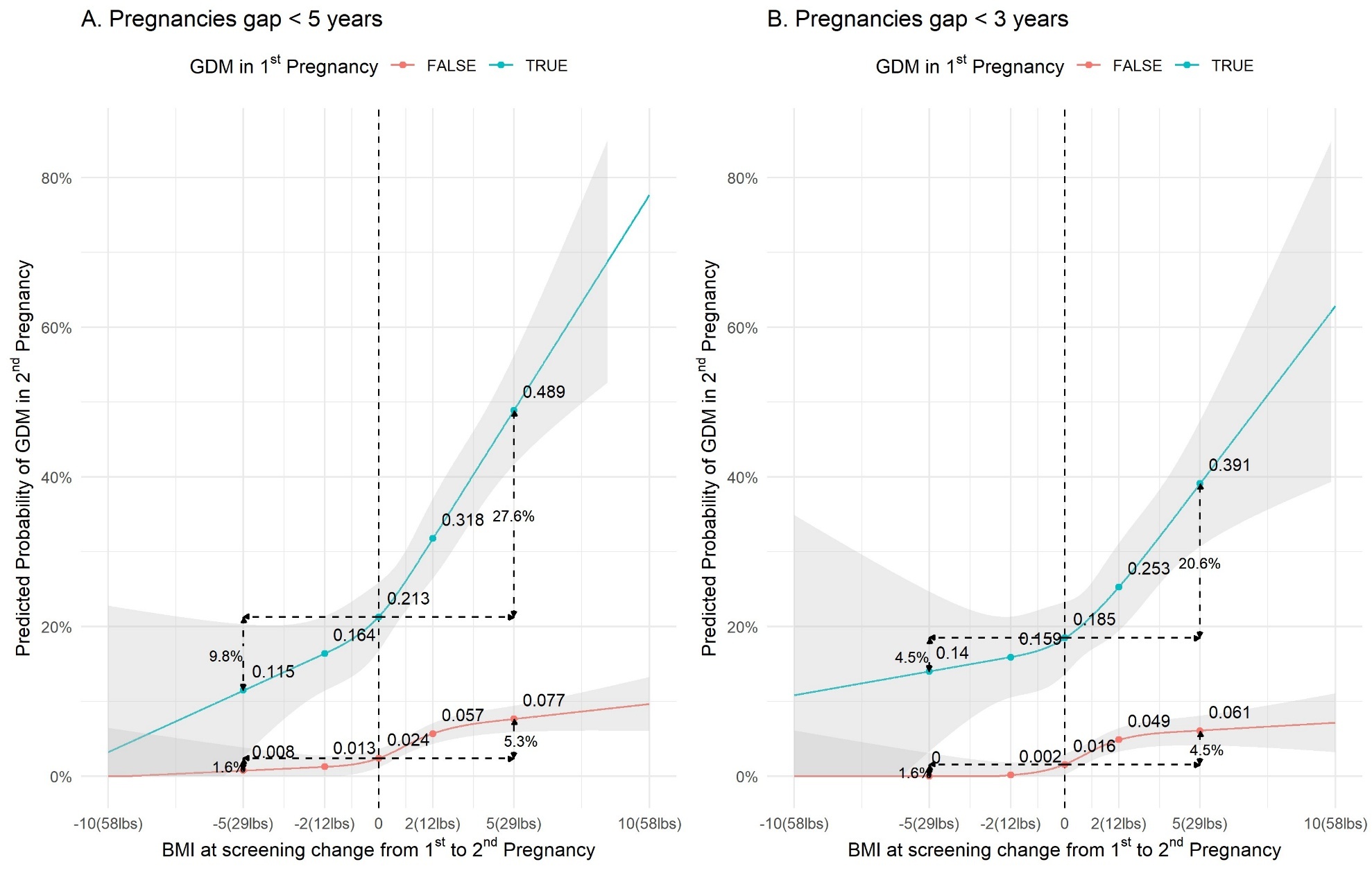


## **Supplementary Figure 3. Predicted probability of GDM in the second recorded pregnancy associated with interpregnancy weight change from first to second recorded pregnancies stratified by GDM status at screening in the first recorded pregnancy among individuals whose GDM screening visit in both pregnancies were done at >=24 weeks of pregnancy.**


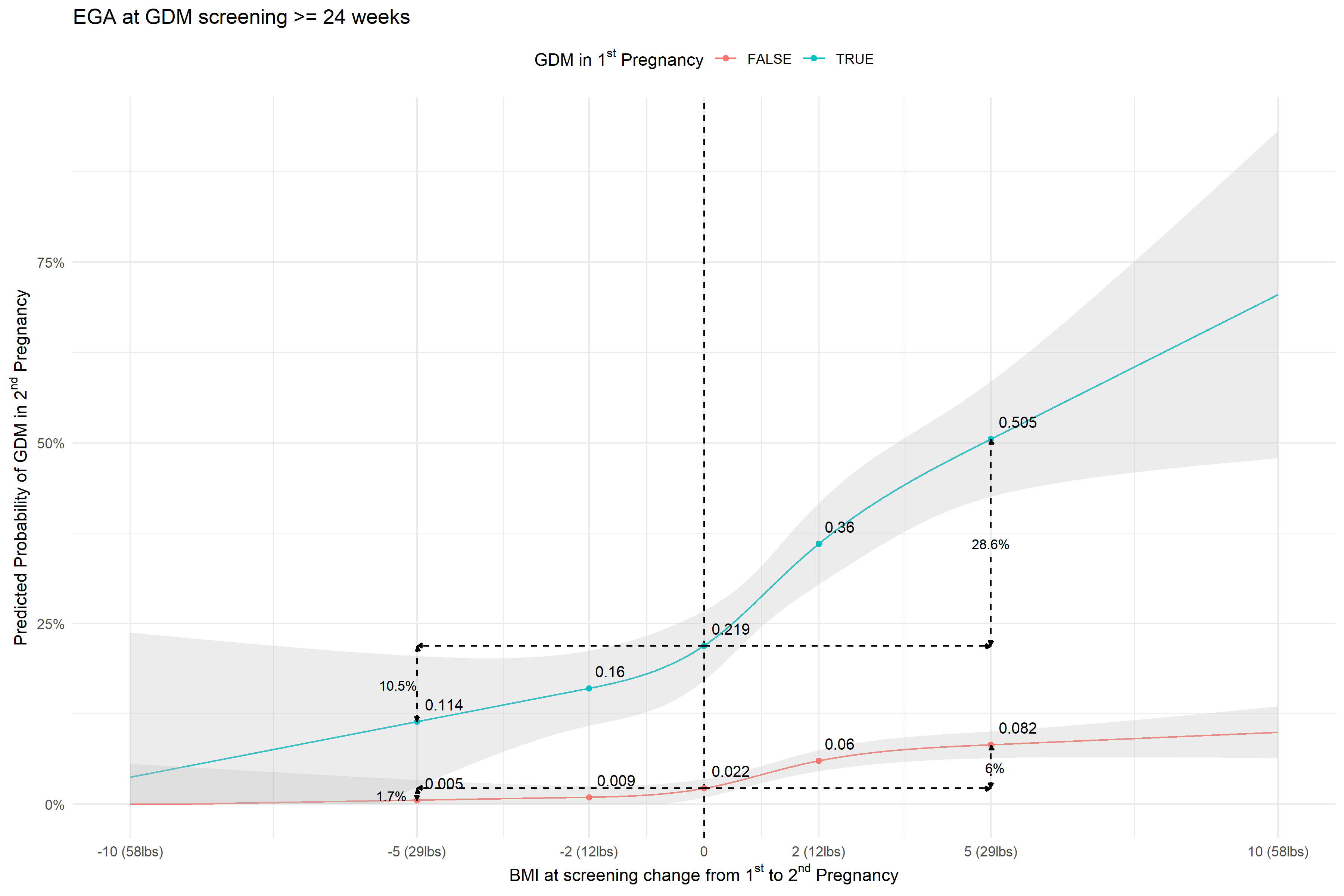


## **Supplementary Figure 4. Predicted probability of gestational diabetes mellitus (GDM) across various races-ethnicities in the second-recorded pregnancy associated with change in body mass index from first to second recorded pregnancies stratified by GDM status at screening in the first-recorded pregnancy**


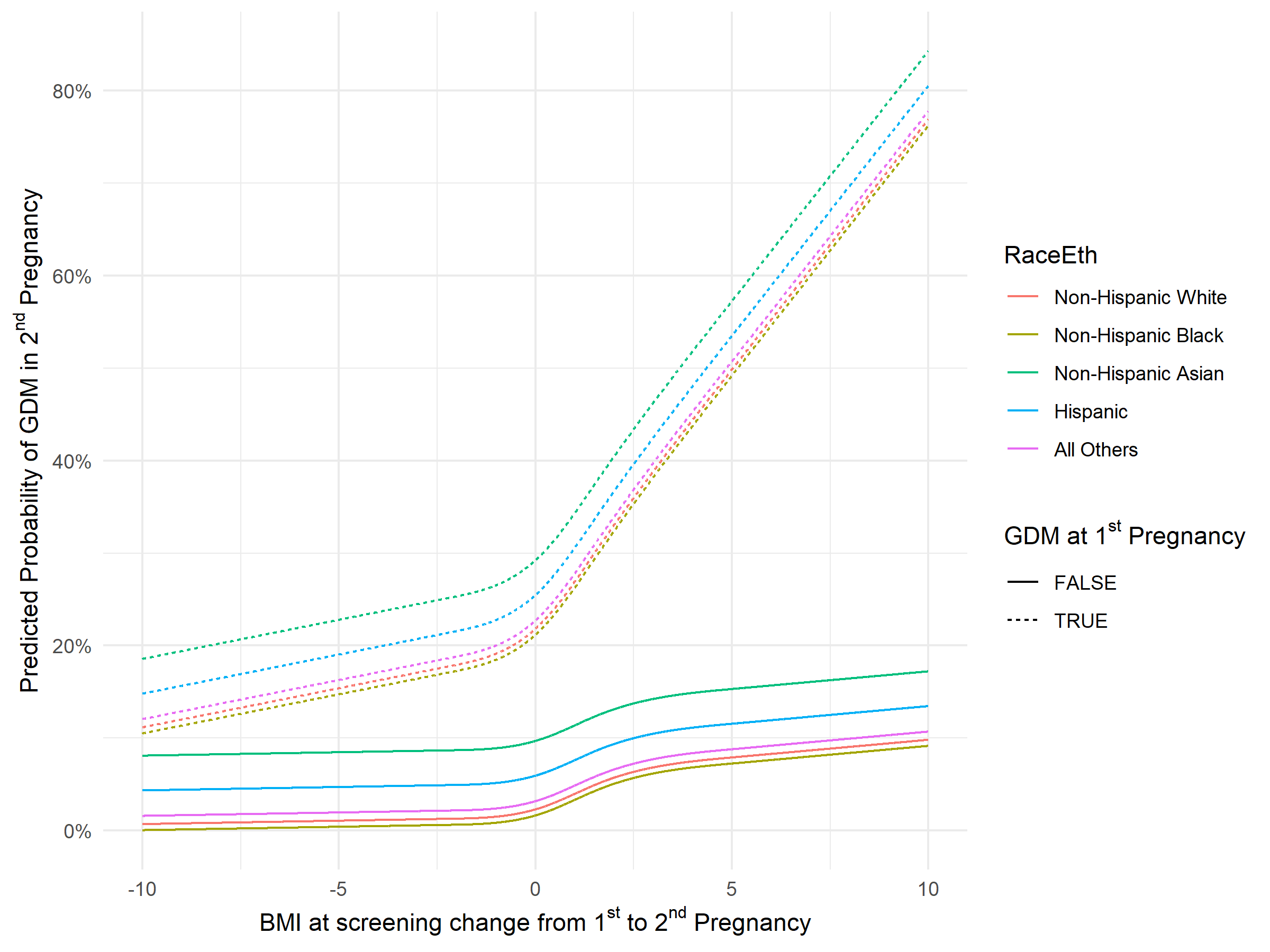


## **Supplementary Figure 5. Predicted probability of GDM in the second recorded pregnancy associated with interpregnancy weight change from first to second recorded pregnancies fixing BMI in the first pregnancy at 20, 30, and 35 kg/m^2^ while stratified by GDM status in the first pregnancy**


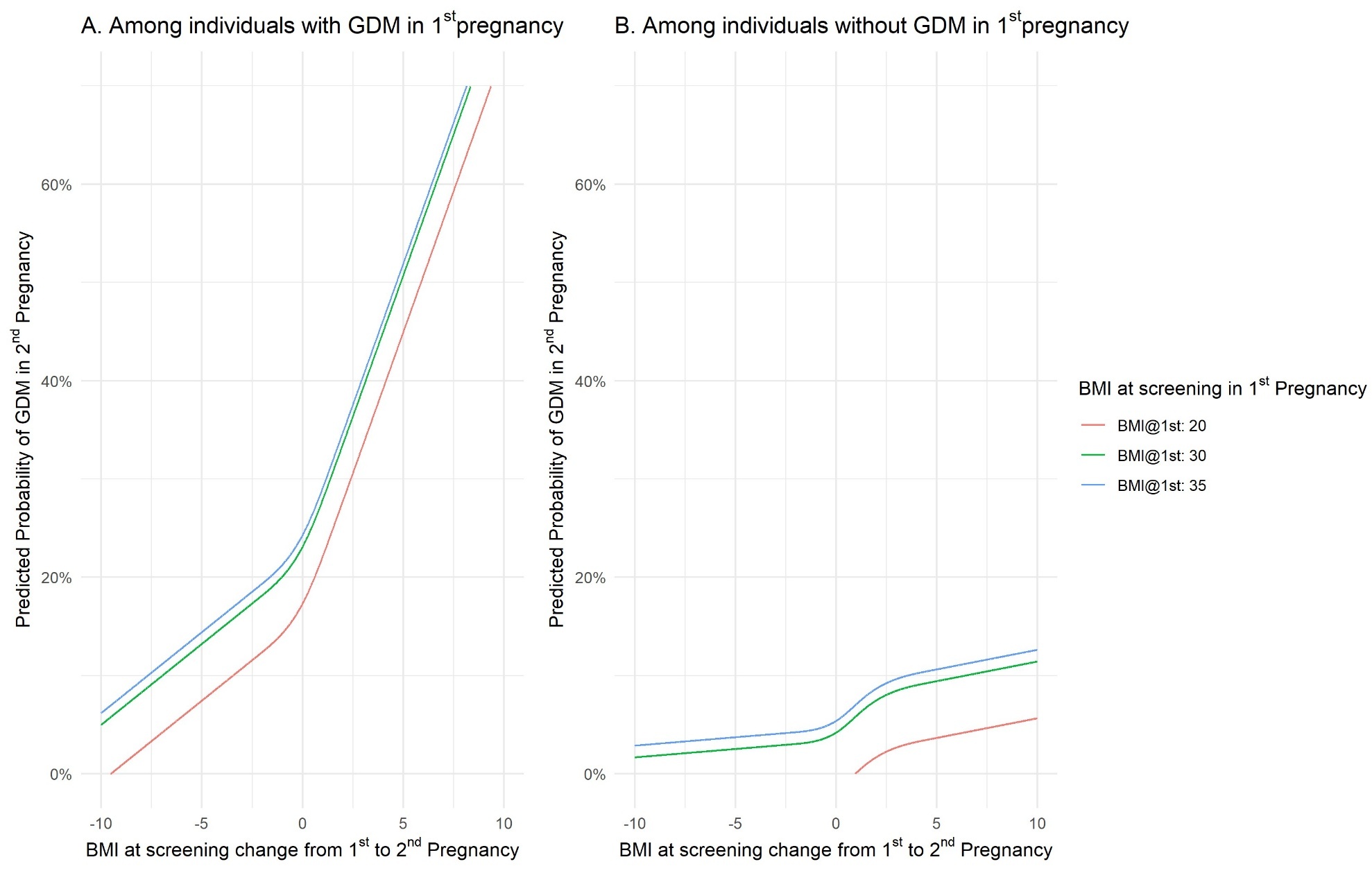


# **SUPPLEMENTARY TABLES**

## **Supplementary Table 1. Demographic characteristics of participants stratified by GDM status in the first- and second-recorded pregnancies**

| **Characteristics** | **Total**  **(n, %)** | **GDM in first pregnancy** | | **GDM in second pregnancy** | |
| --- | --- | --- | --- | --- | --- |
|  |  | **No (n, %)** | **Yes (n, %)** | **No (n, %)** | **Yes (n, %)** |
| **Ethnicity/race** | 4574 | 4372 (96%) | 202 (4%) | 4296 (94%) | 278 (6%) |
| Non-Hispanic White | 2997 (65.5%) | 2867 (95.7%) | 130 (4.3%) | 2843 (94.9%) | 154 (5.1%) |
| Non-Hispanic Black | 663 (14.5%) | 648 (97.7%) | 15 (2.3%) | 633 (95.5%) | 30 (4.5%) |
| Non-Hispanic Asian | 281 (6.1%) | 255 (90.7%) | 26 (9.3%) | 245 (87.2%) | 36 (12.8%) |
| Hispanic | 488 (10.7%) | 463 (94.9%) | 25 (5.1%) | 439 (90.0%) | 49 (10.0%) |
| Others | 145 (3.2%) | 139 (95.9%) | 6 (4.1%) | 136 (93.8%) | 9 (6.2%) |
| **Age at GDM screening** | **Median (Interquartile range)** | | | | |
| For 1^st^ Pregnancy | 27.2 (23.2, 30.6) | 27.2 (23.1, 30.5) | 28.6 (25.2, 32.4) | 27.2 (23.1, 30.5) | 28.1 (24.3, 31.7) |
| For 2^nd^ Pregnancy | 30.1 (26.1, 33.3) | 30.0 (26.1, 33.2) | 31.3 (27.9, 35.5) | 30.0 (26.1, 33.2) | 31.6 (27.5, 35.0) |
| **Estimated gestational age** |  |  |  |  |  |
| For 1^st^ Pregnancy | 28.3 (25.9, 29.4) | 28.3 (25.9, 29.4) | 27.7 (25.2, 29.4) | 28.3 (25.9, 29.4) | 28.0 (25.6, 29.4) |
| For 2^nd^ Pregnancy | 27.1 (25.0, 29.4) | 27.1 (25.0, 29.4) | 27.0 (24.3, 29.4) | 27.1 (25.0, 29.4) | 26.5 (24.6, 29.3) |
| **BMI at GDM screening** |  |  |  |  |  |
| For 1^st^ Pregnancy | 27.2 (24.4, 31.3) | 27.1 (24.4, 31.0) | 30.3 (26.1, 34.7) | 27.0 (24.3, 31.0) | 29.7 (26.4, 33.4) |
| For 2^nd^ Pregnancy | 28.2 (25.0, 32.7) | 28.1 (25.0, 32.5) | 31.1 (26.4, 35.6) | 28.0 (24.9, 32.3) | 31.9 (28.5, 36.1) |
| **Interpregnancy time gap** | 2.4 (1.8, 3.4) | 2.4 (1.8, 3.4) | 2.4 (1.7, 3.3) | 2.4 (1.8, 3.3) | 2.9 (1.9, 4.1) |
| **Interpregnancy weight change [BMI (kg/m^2^)]** | 0.9 (-0.3, 2.2) | 0.9 (-0.2, 2.2) | 0.6 (-0.9, 2.2) | 0.8 (-0.3, 2.1) | 1.8 (0.3, 3.6) |

GDM: Gestational diabetes mellitus; BMI: Body mass index

Note: Interpregnancy weight change indicates the differences between BMI at GDM screening in first and second recorded pregnancies

## **Supplementary Table 2. Clinical and demographic characteristics of participants stratified by categorical exposure status, namely weight loss, no change, and weight gain.**

|  | **No GDM _During_1^st^_Pregnancy_** | | | **GDM _During_1^st^_Pregnancy_** | | | **Overall** | | |
| --- | --- | --- | --- | --- | --- | --- | --- | --- | --- |
|  | **Stable Weight (N=1748)** | **Weight Gain (N=2059)** | **Weight Loss (N=565)** | **Stable Weight (N=71)** | **Weight Gain (N=82)** | **Weight Loss (N=49)** | **Stable Weight (N=1819)** | **Weight Gain (N=2141)** | **Weight Loss (N=614)** |
| **Race/Ethnicity** | |  |  |  |  |  |  |  |  |
| Non-Hispanic White | 1240 (70.9%) | 1223 (59.4%) | 404 (71.5%) | 43 (60.6%) | 52 (63.4%) | 35 (71.4%) | 1283 (70.5%) | 1275 (59.6%) | 439 (71.5%) |
| Non-Hispanic Black | 195 (11.2%) | 370 (18.0%) | 83 (14.7%) | 4 (5.6%) | 8 (9.8%) | 3 (6.1%) | 199 (10.9%) | 378 (17.7%) | 86 (14.0%) |
| Non-Hispanic Asian | 105 (6.0%) | 123 (6.0%) | 27 (4.8%) | 11 (15.5%) | 10 (12.2%) | 5 (10.2%) | 116 (6.4%) | 133 (6.2%) | 32 (5.2%) |
| Hispanic | 155 (8.9%) | 267 (13.0%) | 41 (7.3%) | 9 (12.7%) | 11 (13.4%) | 5 (10.2%) | 164 (9.0%) | 278 (13.0%) | 46 (7.5%) |
| Others | 53 (3.0%) | 76 (3.7%) | 10 (1.8%) | 4 (5.6%) | 1 (1.2%) | 1 (2.0%) | 57 (3.1%) | 77 (3.6%) | 11 (1.8%) |
| **Age at GDM screening** | |  |  |  |  |  |  |  |  |
| During 1^st^ Pregnancy | 28.0 [24.5, 31.0] | 26.2 [22.1, 29.9] | 27.4 [23.6, 30.7] | 29.1 [26.6, 32.9] | 28.0 [25.2, 31.7] | 28.4 [24.6, 31.1] | 28.0 [24.5, 31.1] | 26.3 [22.3, 29.9] | 27.5 [23.6, 30.8] |
| During 2^nd^Pregnancy | 30.6 [26.9, 33.6] | 29.6 [25.4, 33.0] | 30.0 [25.9, 33.3] | 32.1 [29.3, 35.4] | 30.4 [27.6, 35.4] | 31.3 [26.8, 34.5] | 30.7 [27.0, 33.6] | 29.6 [25.5, 33.0] | 30.2 [26.0, 33.3] |
| **Estimated gestational age** | | |  |  |  |  |  |  |  |
| During 1^st^ Pregnancy | 28.4 [26.1, 29.4] | 28.1 [25.7, 29.4] | 29.4 [26.4, 29.4] | 27.9 [24.9, 29.4] | 27.9 [25.4, 29.4] | 28.6 [26.9, 29.4] | 28.4 [26.1, 29.4] | 28.1 [25.7, 29.4] | 29.4 [26.4, 29.4] |
| During 2^nd^Pregnancy | 27.4 [25.0, 29.4] | 27.1 [25.1, 29.4] | 27.1 [24.9, 29.4] | 27.0 [24.8, 29.4] | 27.5 [24.6, 29.4] | 29.1 [25.6, 29.4] | 27.3 [25.0, 29.4] | 27.1 [25.1, 29.4] | 27.3 [24.9, 29.4] |
| **BMI at GDM screening** | |  |  |  |  |  |  |  |  |
| During 1^st^ Pregnancy | 26.1 [23.8, 29.4] | 27.5 [24.6, 31.5] | 29.1 [25.9, 33.0] | 29.2 [25.4, 34.3] | 30.2 [25.8, 34.8] | 31.8 [26.9, 34.7] | 26.2 [23.9, 29.6] | 27.6 [24.6, 31.7] | 29.3 [26.0, 33.3] |
| During 2^nd^Pregnancy | 26.1 [23.9, 29.5] | 30.4 [27.1, 34.9] | 26.7 [24.0, 30.6] | 29.2 [25.3, 34.1] | 33.3 [28.3, 37.6] | 29.2 [24.6, 32.1] | 26.2 [23.9, 29.7] | 30.5 [27.1, 35.0] | 26.8 [24.0, 31.0] |
| **Interpregnancy age gap** | |  |  |  |  |  |  |  |  |
| Median [Q1, Q3] | 2.3 [1.8, 3.0] | 2.6 [1.8, 3.8] | 2.2 [1.7, 3.0] | 2.3 [1.9, 3.1] | 2.7 [1.6, 3.6] | 2.5 [1.7, 3.4] | 2.3 [1.8, 3.0] | 2.6 [1.8, 3.8] | 2.2 [1.7, 3.0] |
| **BMI Change (continuous)** | |  |  |  |  |  |  |  |  |
| Median [Q1, Q3] | 0.1 [-0.3, 0.5] | 2.3 [1.6, 3.6] | -1.8 [-2.5, -1.3] | 0.1 [-0.4, 0.7] | 2.4 [1.8, 3.0] | -2.4 [-3.1, -1.7] | 0.1 [-0.3, 0.5] | 2.3 [1.6, 3.6] | -1.9 [-2.6, -1.4] |
| **GDM Status _During_2^nd^_Pregnancy_** | | | | | | | | | |
| Yes | 54 (3.1%) | 148 (7.2%) | 15 (2.7%) | 19 (26.8%) | 32 (39.0%) | 10 (20.4%) | 73 (4.0%) | 180 (8.4%) | 25 (4.1%) |
| No | 1694 (96.9%) | 1911 (92.8%) | 550 (97.3%) | 52 (73.2%) | 50 (61.0%) | 39 (79.6%) | 1746 (96.0%) | 1961 (91.6%) | 589 (95.9%) |

GDM: Gestational Diabetes Mellitus; BMI: Body Mass Index

## **Supplementary Table 3. Comparison of patient characteristics: excluded vs. analyzed data sets based on the availability of screening information**

|  | **Excluded (N=24475)** | **Included (N=4574)** |
| --- | --- | --- |
| **Race/Ethnicity** | | |
| Non-Hispanic White | 14296 (58.4%) | 2997 (65.5%) |
| Non-Hispanic Black | 4609 (18.8%) | 663 (14.5%) |
| Non-Hispanic Asian | 939 (3.8%) | 281 (6.1%) |
| Hispanic | 2552 (10.4%) | 488 (10.7%) |
| All Others | 2079 (8.5%) | 145 (3.2%) |
| **Age at GDM screening**during 1^st^ Pregnancy | | |
| Median [Q1, Q3] | 27.1 [22.4, 31.3] | 27.3 [23.2, 30.6] |
| Missing | 13615 (55.6%) | 0 (0%) |
| **Age at GDM screening**during 2^nd^ Pregnancy | | |
| Median [Q1, Q3] | 28.6 [24.0, 32.8] | 30.1 [26.1, 33.3] |
| Missing | 16274 (66.5%) | 0 (0%) |
| **Estimated gestational age** during 1^st^ Pregnancy | | |
| Median [Q1, Q3] | 28.0 [25.6, 29.4] | 28.4 [26.0, 29.4] |
| Missing | 13615 (55.6%) | 0 (0%) |
| **Estimated gestational age** during 2^nd^ Pregnancy | | |
| Median [Q1, Q3] | 27.9 [25.4, 29.4] | 27.3 [25.0, 29.4] |
| Missing | 16274 (66.5%) | 0 (0%) |
| **BMI at GDM screening**during 1^st^ Pregnancy | | |
| Median [Q1, Q3] | 28.1 [25.0, 32.8] | 27.1 [24.4, 31.1] |
| Missing | 17173 (70.2%) | 0 (0%) |
| **BMI at GDM screening**during 2^nd^ Pregnancy | | |
| Median [Q1, Q3] | 28.8 [25.3, 33.9] | 28.2 [25.0, 32.6] |
| Missing | 18356 (75.0%) | 0 (0%) |
| **Interpregnancy age gap** | | |
| Median [Q1, Q3] | 2.4 [1.6, 3.6] | 2.4 [1.8, 3.4] |
| Missing | 21157 (86.4%) | 0 (0%) |
| **BMI Change (continuous)** | | |
| Median [Q1, Q3] | 0.5 [-1.1, 2.0] | 0.9 [-0.3, 2.2] |
| Missing | 23578 (96.3%) | 0 (0%) |

GDM: Gestational Diabetes Mellitus; BMI: Body Mass Index

## **Supplementary Table 4. Comparison of patient characteristics: excluded vs. analyzed data sets based on BMI information**

|  | **Excluded individuals lacking BMI information for either pregnancy (N = 2,549)** | | |  | **Analyzed data with BMI information for both pregnancies (N = 4,574)** | | |
| --- | --- | --- | --- | --- | --- | --- | --- |
|  | **GDM in second pregnancy** | | |  | **GDM in second pregnancy** | | |
|  | **No** | **Yes** | **Total** |  | **No** | **Yes** | **Total** |
|  | **n (%) or Median (IQR)** | | |  | **n (%) or Median (IQR)** | | |
| **GDM status at first pregnancy** | |  |  |  |  |  |  |
| No | 2,354 (96.2%) | 94 (3.8%) | 2,448 (96.0%) |  | 4,155 (95.0%) | 217 (5.0%) | 4372 (95.6%) |
| Yes | 71 (70.3%) | 30 (29.7%) | 101 (4.0%) |  | 141 (69.8%) | 61 (30.2%) | 202 (4.4%) |
| **Ethnicity/Race** |  |  |  |  |  |  |  |
| Non-Hispanic White | 1,407 (95.7%) | 63 (4.3%) | 1470 (57.7%) |  | 2843 (94.9%) | 154 (5.1%) | 2997 (65.5%) |
| Non-Hispanic Black | 606 (95.9%) | 9 (8.3%) | 632 (24.8%) |  | 633 (95.5%) | 30 (4.5%) | 663 (14.5%) |
| Hispanic | 213 (91.8%) | 19 (8.2%) | 232 (9.1%) |  | 245 (87.2%) | 36 (12.8%) | 281 (6.1%) |
| Non-Hispanic Asian | 99 (91.7%) | 9 (8.3%) | 108 (4.2%) |  | 439 (90.0%) | 49 (10.0%) | 488 (10.7%) |
| All Others | 100 (93.5%) | 7 (6.5%) | 107 (4.2%) |  | 136 (93.8%) | 9 (6.2%) | 145 (3.2%) |
| **Age at screening (Year)** | 28.8 (23.9, 33.0) | 31.1 (27.5, 34.2) | 28.9 (24.0, 33.1) |  | 30.0 (26.1, 33.2) | 31.6 (27.5, 35.0) | 30.1 (26.1, 33.3) |
| **Estimated gestational age (Week)** | 28.0 (25.7, 29.4) | 27.0 (24.7, 29.4) | 27.9 (25.7, 29.4) |  | 27.1 (25.0, 29.4) | 26.5 (24.6, 29.3) | 28.3 (25.9, 29.4) |
| **BMI at 1^st^ pregnancy screening (kg/m^2^) ^a^** | 26.9 (24.3, 31.2) | 31.2 (27.6, 34.7) | 27.1 (24.4, 31.7) |  | 27.0 (24.3, 31.0) | 29.7 (26.4, 33.4) | 27.2 (24.4, 31.3) |
| **BMI at 2^nd^ pregnancy screening (kg/m^2^) ^b^** | 28.2 (25.1, 33.1) | 33.3 (28.0, 38.9) | 28.3 (25.3, 33.4) |  | 28.0 (24.9, 32.3) | 31.9 (28.5, 36.1) | 28.2 (25.0, 32.7) |

GDM: Gestational Diabetes Mellitus; BMI: Body Mass Index; IQR: Interquartile range

**^a^**: BMI information was available only for 523 individuals at 1^st^ pregnancy out of 2,549 excluded individuals.

**^b^:** BMI information was available only for 1,242 individuals at 2^nd^ pregnancy out of 2,549 excluded individuals.
